# A hiatus in the rivalry between Pierre Marie and Jules Dejerine: a collaborative study on sensory disorders by Andre Pierre Marie and Gustave Roussy

**DOI:** 10.1055/s-0044-1788270

**Published:** 2024-07-18

**Authors:** Carlos Henrique Ferreira Camargo, Emanuel Cassou, Francisco Manoel Branco Germiniani, Hélio Afonso Ghizoni Teive

**Affiliations:** 1 Universidade Federal do Paraná, Programa de Pós-Graduação em Medicina, Curitiba PR, Brazil.; 2 Universidade Federal do Paraná, Departamento de Clínica Médica, Serviço de Neurologia, Curitiba PR, Brazil.

**Keywords:** History of Medicine, Neurology, Thalamic Diseases, Pain, Hypesthesia, Agnosia, História da Medicina, Neurologia, Doenças Talâmicas, Dor, Hipestesia, Agnosia

## Abstract

Personal and professional rivalries involving prominent neurologists mark the history of nineteenth-century French neurology. One of the great examples is the feud between Pierre Marie and Jules Dejerine. The dispute between the two, nevertheless, did not prevent Pierre Marie's son, André Marie, and Gustave Roussy – one of Dejerine's favorite pupils, from collaborating on significant research that led to the doctoral dissertation by Andre Marie regarding sensory disturbances associated with painful hemiagnosia found in thalamic lesions.

## Resumo

As rivalidades pessoais e profissionais entre neurologistas proeminentes marcaram a história da neurologia francesa do século XIX. Um dos grandes exemplos é a rivalidade entre Pierre Marie e Jules Dejerine. A disputa entre os dois, no entanto, não impediu que o filho de Pierre Marie, André Marie, e Gustave Roussy, um dos pupilos preferidos de Dejerine, colaborassem numa investigação significativa que resultou na tese de doutorado de André Marie sobre os distúrbios sensoriais associados à hemiagnosia dolorosa encontrada nas lesões talâmicas.

## INTRODUCTION

In the late nineteenth and early twentieth centuries, Pierre Marie (1853–1940), one of Jean-Martin Charcot's (1825–1893) most devoted disciples, and Jules Dejerine (1849–1917), a disciple of Alfred Vulpian (1826–1887), made pivotal contributions to the development of neurology. At that time, the French School of Neurology was recognized worldwide as one of the most influential in the world. 1 Due to his outstanding scientific production, Dejerine emerged as a solid intellectual force at Bicêtre Hospital, becoming a rival and eventually an adversary of the Charcot's school at La Salpêtrière Hospital. 2

Pierre Marie and Dejerine engaged in several intense scientific confrontations. For example, an intellectual duel, later known as the Paris “aphasia debate,” occurred in 1908. Another, which nearly resulted in a real-life duel, occurred in 1893. 1 The clash reached a critical point after Pierre Marie's publication on sensory ataxia 3 faced severe criticism from Dejerine in another article, 4 followed by a vehement counterattack by Marie. Dejerine subsequently sent emissaries to Marie's house, summoning him to retract or face a death duel, allowing the choice of location and date. The witnesses acted swiftly to reconcile the two adversaries, thus averting the potentially fateful duel. 1

In 1893, tensions escalated after Charcot's death in the intense competition for his succession to the Chair of Diseases of the Nervous System at La Salpêtriére. After an interim period under Édouard Brissaud's (1852–1909) leadership and a subsequent tenure under Fulgence Raymond (1844–1910), a new public competition was instituted, and Dejerine was appointed to the position in 1910. In 1907, Pierre Marie became the Chair of Anatomical Pathology at the School of Medicine, and, following Dejerine's death in 1917, he achieved the Chair of Diseases of the Nervous System at La Salpêtriére through a public competition, retiring in 1925. 1 5 Upon his return to La Salpêtrière, he promptly ordered the expulsion of Dejerine's widow, Augusta Dejerine-Klumpke (1859–1927), also a formidable neurologist, from the hospital, granting 2 weeks for this action to be carried out. 5

The sagas of families who perpetuate patriarchal feuds are well documented. The purpose of this historical report is to highlight an exception. Andre Henri Pierre Marie (1891–1929), son of Pierre Marie, enlisted the support of Gustave Roussy (1874–1948), one of Dejerine's most significant pupils, in his doctoral thesis. 6

### André Pierre Marie

**Figure 1:**
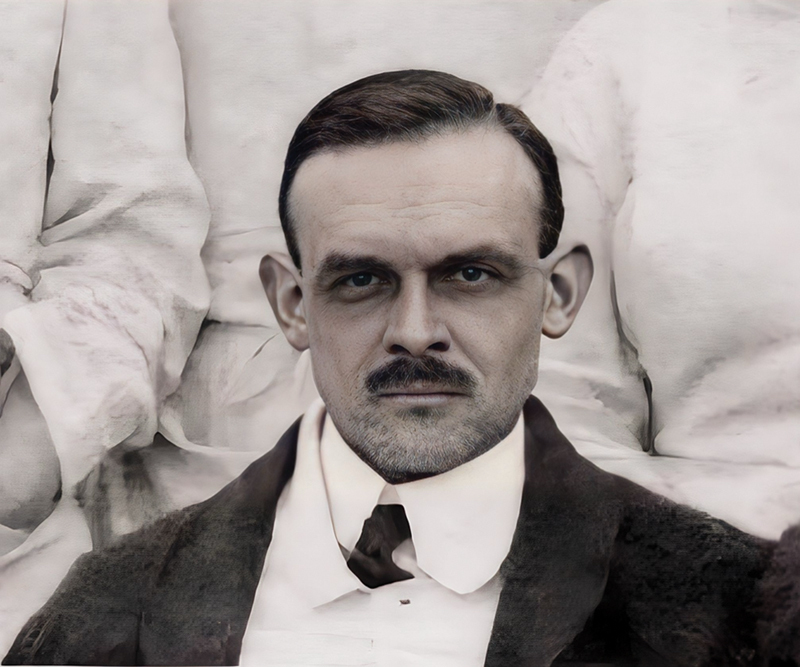
André Henri Pierre Marie (1874–1929). (From the personal archives of Dr. Olivier Walusinski).

André Pierre Marie ( Figure 1 ) was born in Paris and was the second son of Pierre Marie, under whom he interned at La Salpêtriére in 1920. 7 In 1921, André Marie spent a year interning at the Saint-Louis Hospital under the supervision of Georges Thibierge (1856–1926). He returned for 2 years to La Salpêtrière, where he was a resident under his father in the neurological clinic. 7 André Marie defended his doctoral thesis in 1924, entitled “Étude comparée des troubles sensifs d'origine cérébrale, lésions corticales et thalamiques, hémiagnosie douloureuse” 6 to a panel presided by Georges Guillain (1876–1963). Later, he worked at the Pasteur Institute in Paris, focusing on infections and intoxications. Unfortunately, he suffered a premature death in 1929 due to botulism after a drop of botulinum toxin fell into his eye during a laboratory experiment. 7

### Gustave Roussy

**Figure 2:**
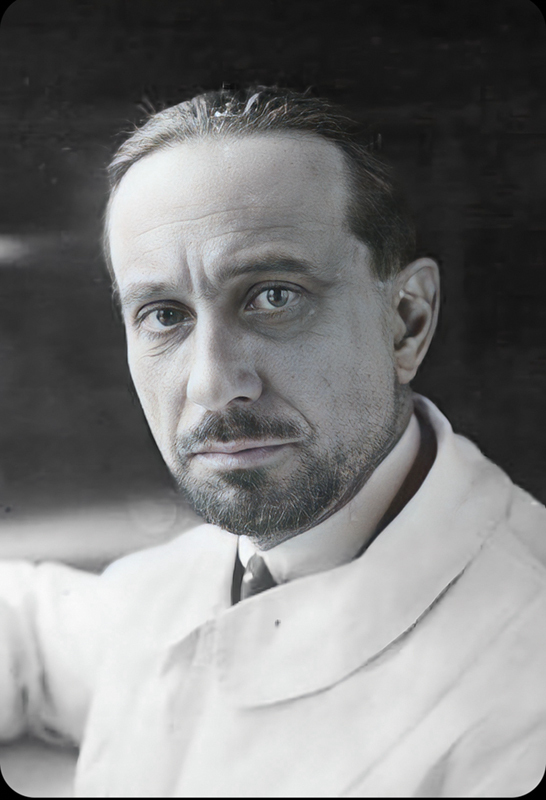
Gustave Roussy (1874–1948). (Extracted from Wikipedia – *Bibliothèque Nationale de France* - September 7th, 2023).

Gustave Roussy (1874–1948) was born in Switzerland and graduated in medicine in Geneva ( Figure 2 ). He later moved to Paris, where he completed his training in neurology and neuropathology and acquired French citizenship. 8 Roussy gained international recognition following the publication of his work in 1906 on the thalamic syndrome, now known as Dejerine-Roussy syndrome. 9 10 11 12 13 His doctoral thesis entitled “La couche optique (Étude anatomique, physiologique & clinique): Le thalamique syndrome” was published in 1907. 11 Despite being one of Dejerine's closest pupils, Roussy collaborated with Pierre Marie since 1907, eventually succeeding him as Chair of Pathological Anatomy upon his retirement. 8 13 Pierre Marie was a very influential mentor for Roussy's career, especially for his teachings on pathology. They published clinical cases, including a collaboration on cholesteatoma. 14 In 1925, Roussy played a pivotal role in establishing a center for the study and care of cancer patients in the suburbs of Paris, which now bears his name. While dean of the School of Medicine in Paris (1933–1937), he was elected rector of the University in 1937. Still, in 1940, he was removed from office because he supported the students participating in the resistance to the Nazi occupation. Roussy committed suicide in 1948 and was laid to rest in the Montparnasse cemetery in Paris. 8

### The collaboration of André Pierre Marie and Gustave Roussy

André Marie's thesis aimed to differentiate sensory deficits of cortical origin from those in the thalamus. 6 The thalamic syndrome, previously defined by Dejerine and Roussy, was characterized by the presence of several elements:

- mild hemiparesis;
- persistent superficial hemianesthesia, sometimes associated with cutaneous hyperesthesia;
- mild hemiataxia, sometimes associated with astereognosia;
- persistent, intense, and intolerable paroxysmal pain on the hemiparetic side; and
- choreoathetotic movements in the limbs ipsilateral to the motor deficit. 10 11

Similarly, in 1915, Dejerine and Jean Mouzon (1892–1964) described a clinical parietal syndrome, termed *cortical sensory syndrome* or Dejerine-Mouzon syndrome. This syndrome is characterized by touch, temperature, and pain hemianesthesia associated with anesthesia-induced hemiparesis and astereognosis. 15 16 Another syndrome described in 1900 was the Verger-Dejerine syndrome, which involved sensory deficits and the inability to identify a familiar object through palpation. 17

André Marie's thesis had the collaboration of his advisor Henri Bouttier (1888–1923), his father Pierre Marie, and the significant participation of Gustave Roussy, 6 7 18 19 20 studied various sensitive disorders, sometimes in association with motor deficits, hemianopsia, and athetoid movements. The thesis delved into the dissociated thalamic syndrome (impairment of superficial sensitivity with preservation of deep sensitivity), the global thalamic syndrome, and, notably, the definition of painful hemiagnosia (inability to discern the quality and point of application of painful stimulation) in patients with recent hemiplegia. 6 18 19 20 According to André Marie's descriptions, pain conditions are associated with thalamic involvement, while cortical (parietal) sensory deficits are painless. 6

In conclusion, despite the significant personal animosity and scientific rivalry between Pierre Marie and Jules Dejerine, André Marie and Gustave Roussy engaged in a highly productive scientific collaboration. This collaboration resulted in the publication of Andre Marie's doctoral thesis in 1924, describing a crucial study on sensory disorders associated with cortical and thalamic brain lesions, focusing on painful hemiagnosia.
